# Evaluation of a Digital Previsit Tool for Identifying Stroke-Related Health Problems Before a Follow-Up Visit (Part 1): Survey Study

**DOI:** 10.2196/55852

**Published:** 2024-09-03

**Authors:** Petra Pohl, Karoline Klerfors, Emma K Kjörk

**Affiliations:** 1 Department of Health and Rehabilitation Institute of Neuroscience and Physiology Sahlgrenska Academy, University of Gothenburg Gothenburg Sweden; 2 Department of Clinical Neuroscience Institute of Neuroscience and Physiology Sahlgrenska Academy, University of Gothenburg Gothenburg Sweden; 3 Centre for Person-Centred Care (GPCC) University of Gothenburg Gothenburg Sweden; 4 Sahlgrenska University Hospital Gothenburg Sweden

**Keywords:** e-health, stroke, Strokehälsa, follow-up, previsit, person-centred care, health literacy, digital tool, shared decision-making, survey, mobile phone

## Abstract

**Background:**

Stroke may lead to various disabilities, and a structured follow-up visit is strongly recommended within a few months after an event. To facilitate this visit, the digital previsit tool “Strokehealth” was developed for patients to fill out in advance. The concept Strokehälsa (or Strokehealth) was initially developed in-house as a Windows application, later incorporated in 1177.se.

**Objective:**

The study’s primary objective was to use a patient satisfaction survey to evaluate the digital previsit tool Strokehealth when used before a follow-up visit, with a focus on feasibility and relevance from the perspective of people with stroke. Our secondary objective was to explore the extent to which the previsit tool identified stroke-related health problems.

**Methods:**

Between November 2020 and June 2021, a web-based survey was sent to patients who were scheduled for a follow-up visit after discharge from a stroke unit and had recently filled in the previsit tool. The survey covered demographic characteristics, internet habits, and satisfaction rated using 5 response options. Descriptive statistics were used to present data from both the previsit tool and the survey. We also compared the characteristics of those who completed the previsit tool and those who did not, using nonparametric statistics. Free-text responses were thematically analyzed.

**Results:**

All patients filling out the previsit tool (80/171; age: median 67, range 32-91 years) were community-dwelling. Most had experienced a mild stroke and reported a median of 2 stroke-related health problems (range 0-8), and they were significantly younger than nonresponders (*P*<.001). The survey evaluating the previsit tool was completed by 73% (58/80; 39 men). The majority (48/58, 83%) reported using the internet daily. Most respondents (56/58, 97%) were either satisfied (n=15) or very satisfied (n=41) with how well the previsit tool captured their health problems. The highest level of dissatisfaction was related to the response options in Strokehealth (n=5). Based on the free-text answers to the survey, we developed 4 themes. First, Strokehealth was perceived to provide a structure that ensured that issues would be emphasized and considered. Second, user-friendliness and accessibility were viewed as acceptable, although respondents suggested improvements. Third, participants raised awareness about being approached digitally for communication and highlighted the importance of how to be approached. Fourth, their experiences with Strokehealth were influenced by their perceptions of the explanatory texts, the response options, and the possibility of elaborating on their answers in free text.

**Conclusions:**

People with stroke considered the freely available previsit tool Strokehealth feasible for preparing in advance for a follow-up visit. Despite high satisfaction with how well the tool captured their health problems, participants indicated that additional free-text responses and revised information could enhance usability. Improvements need to be considered in parallel with qualitative data to ensure that the tool meets patient needs.

**Trial Registration:**

Researchweb 275135; https://www.researchweb.org/is/vgr/project/275135

## Introduction

Stroke affects more than 101 million people worldwide [1] and can lead to a range of physical, cognitive, and emotional disabilities [2]. In its newly launched “Package of interventions for rehabilitation for stroke” within its “Rehabilitation 2030 initiative,” the World Health Organization makes clear that people living with stroke need lifelong access to rehabilitation services because of continuing health problems [3]. Furthermore, there is a general trend toward shorter hospital stays after a stroke. Consequently, it is vital to identify individuals at risk of secondary health issues to provide them with prevention, treatment, and support in developing self-management strategies. Continuous health care interactions are required to achieve these outcomes [4].

Depending on the problem area, patients should be assessed accordingly to access targeted interventions including education, advice, and support for self-management [3]. Postdischarge stroke care, however, is often fragmented without standardized routines for long-term follow-up [4], and people frequently have difficulties accessing health care [5]. According to the Stroke Action Plan for Europe, structured follow-up visits should be offered to all patients within 6 months after a stroke to identify stroke-related health problems, address rehabilitation needs, and support adaptation to life after a stroke [4].

As part of the structures of care for people with long-term conditions, patients must be duly prepared and sufficiently informed before a visit to a proactive health care team [6]. In addition, a person-centered approach that involves patients in shared decision-making contributes to a positive impact on health outcomes and patient satisfaction [7]. A person-centered approach entails acknowledgment within health care services that individuals can collaborate with health care professionals and actively engage in the decision-making process, which nurtures the patient’s sense of empowerment [8].

To support better-structured follow-up visits after a stroke, the dialogue tool “Post-Stroke Checklist” was developed for health care professionals to use during outpatient visits [9]. The checklist comprises 11 questions that can aid in identifying common health problems (eg, mobility, cognition, and life after stroke) and guide health care professionals on appropriate actions, including recommendations for referrals [9]. Satisfaction with the dialogue tool has generally been high, but health care professionals have noted challenges in managing the checklist within the allotted timeframe [10,11], and patients have requested the ability to prepare in advance [11]. This can be achieved with previsit tools that can enhance patient experience, patient engagement, and practice efficiency [12]. In response to this, the previsit tool Strokehälsa (“Strokehealth”) was developed based on the questions from the Post-Stroke Checklist [13]. The aim was to capture health problems and provide patients with information about common consequences after stroke and time to reflect [13]. Initially designed as a digital tool to enhance accessibility and usefulness, Strokehealth is now also available in a picture-supported version and in paper format, freely accessible in multiple languages. Other digital previsit tools usually concentrate solely on gathering self-reported data [12], or they may be more comprehensive, such as the stroke-related previsit tool “Rehabkompassen” [14]. Both Strokehealth and Rehabkompassen were initially developed in-house as a Windows application and later incorporated in the Swedish national patient portal.

Development and use of digital health service tools are crucial to involving patients in proactive management of their health [15]. Furthermore, as the use of diverse technologies increases within the health care domain, more patient engagement and participation will be required [16]. In general, digital tools yield positive impacts on patient empowerment, self-management, communication, and patient engagement [16-18]. In those with long-term conditions such as stroke, however, digital health solutions must be user-friendly in terms of eHealth literacy needs (ie, ability to comprehend health information and actively engage with eHealth services [19]), as these patients report related difficulties more than the general population [20]. The development of these tools thus must incorporate consideration of patient-related factors, including cognitive ability to process information, need for a sense of security and control, and intrinsic motivation to engage with digital health care services [19]. The patient perspective is a high priority in eHealth [17] because personal motivation for using digital health services is key to gaining eHealth literacy [19]. In keeping with these precepts, development of the previsit tool, Strokehealth, incorporated a comprehensive participatory approach, involving people with first-hand experience with stroke and health care professionals in the co-design process [13].

Initially, a prototype of Strokehealth was created, and subsequent iterations were tested on purposively selected patients with stroke and relevant health care professionals [13]. Based on user feedback, version 1.0 incorporated 11 features from the Post-Stroke Checklist and 3 additional questions pertaining to oral health, eating or swallowing problems, and other challenges after stroke. The final question offered a free-text option for users to add anything else they wished to share before the visit. In addition, explanatory texts were attached to all questions, and an advisory text was added. Version 1.0 was launched at the Swedish national patient portal 1177.se. Personal accounts are created in the patient portal using a social security number and an electronic ID, enabling notifications for activities such as form submissions. We chose this secure platform because it provides a sustainable solution and enhances accessibility for patients [13].

The aim was to develop an easy-to-use previsit tool perceived as meaningful for users, with optimized conditions for implementation in keeping with service-design principles [21]. Strokehealth already has shown a potential to enable people with stroke to prepare for visits, to capture care needs, and to provide patients with valuable information related to stroke [13]. Its validity in a real-world setting, however, remains to be established. Real world feasibility testing is needed to evaluate the tool’s usability and efficacy in capturing health problems and to facilitate its further development. The main study aim, thus, was to evaluate the previsit tool, Strokehealth, as used by people with stroke before a scheduled follow-up visit, with a focus on feasibility and relevance from the patient’s perspective. A second aim was to explore the extent to which Strokehealth could identify stroke-related health problems.

## Methods

### Study Design

This study is part of a larger research project aimed at developing and evaluating the digital previsit tool Strokehealth in an article series with quantitative and qualitative methods, in line with the established framework for developing and evaluating complex interventions [22]. In total, 2 separate data collection procedures were used in this study (part 1)—one from the previsit tool and the other from a subsequently administered closed web-based patient satisfaction survey. The CHERRIES (Checklist for Reporting Results of Internet E-Surveys) checklist was followed in this study (Multimedia Appendix 1) [23]. Web-based surveys provide an efficient way to track user views and allow for the analysis of large volumes of information [24].

### Ethical Considerations

The study was approved by the Swedish Ethical Review Authority (2017/556-17, 2020-03324, and 2021-06723-02). Patients received information about the research project after logging into the patient portal and were asked whether they agreed to participate in the study. Informed consent was obtained from patients as part of the process. It was not possible to continue filling out the survey if this question remained unanswered. The initial information clearly stated that completing the survey was voluntary, and that participation would not affect their medical care. No incentives were offered.

### Study Context

Between November 2020 and June 2021, consecutive patients discharged from a stroke unit and scheduled for a follow-up visit with a stroke team member in primary health care received a digital message instructing them to log in to the patient portal and complete the previsit tool, Strokehealth, before their appointment (within 1-2 weeks). After submitting Strokehealth, patients received a second digital message prompting them to log in to the portal once again, where they were encouraged to fill out the patient satisfaction survey on the same platform (Multimedia Appendix 2). The log-in requirement prevented duplicate entries from the same user so that only unique visitors were registered.

### Sampling and Participants

A convenience sampling was used with the aim of gathering data from a minimum of 50 patients [25]. Furthermore, 4 publicly-funded health care units were invited to participate (3 hospital-based stroke units and 1 primary health care unit). After inclusion in the study, the primary health care unit withdrew from participation because of reorganizations resulting from the COVID-19 pandemic, so ultimately only the 3 hospital units were included. One hospital (unit 1) was situated in an urban area, and 2 (unit 2 and unit 3) were situated in 2 middle-sized cities serving more rural areas. In addition, 1 stroke-specialized nurse at each unit was selected to participate. The follow-up visit was scheduled between 3 weeks and 3 months after the stroke event, depending on the standard routines within each unit.

### Data Collection From the Previsit Tool Strokehealth

After receiving the digital message about Strokehealth, patients could choose to fill out the tool directly or to do so later. Help from next-of-kin was allowed but asked to be noted in the response. Each of the 3 nurses could monitor response status, and if no response was obtained, they could send a total of 2 reminders.

**Figure 1:**
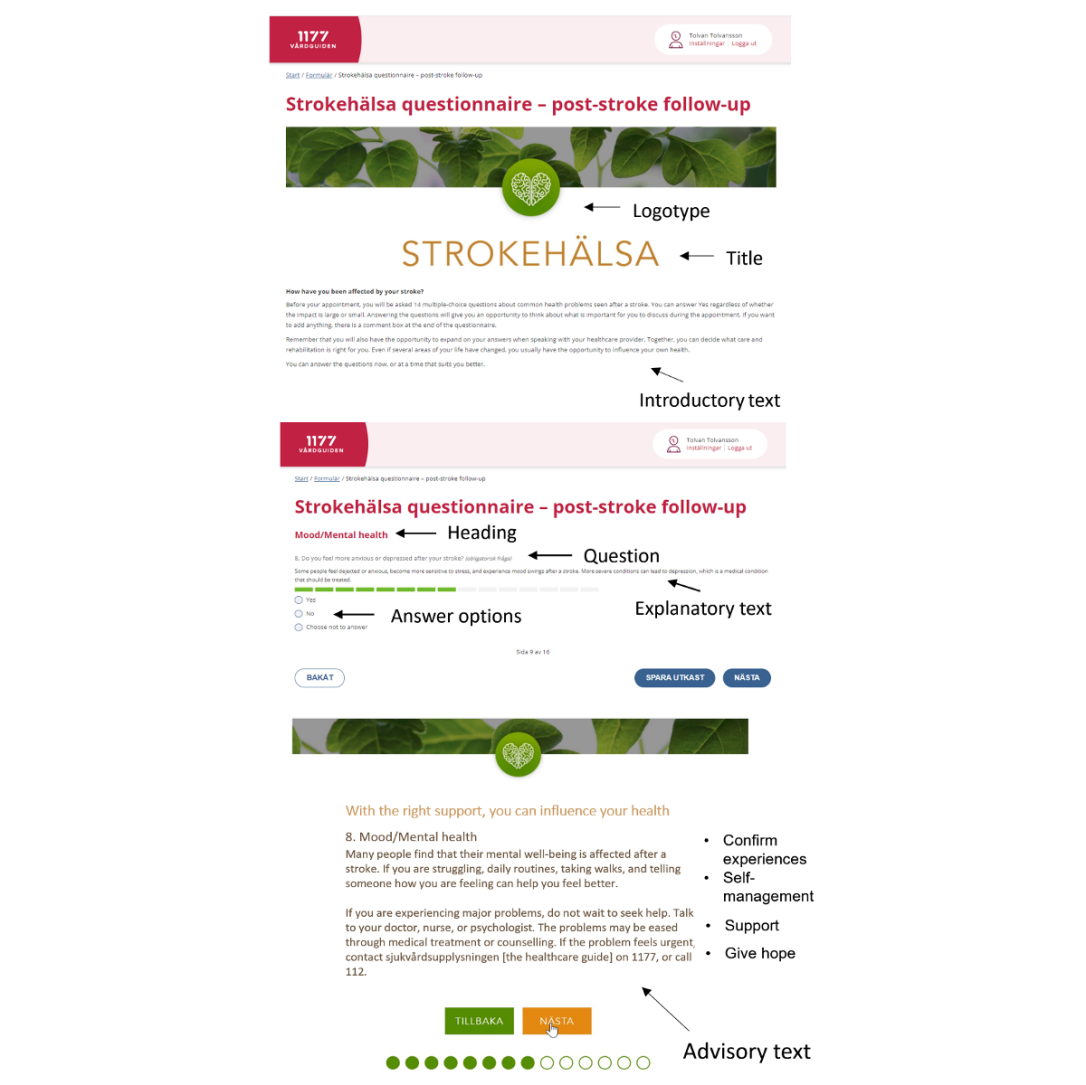
A screenshot of the elements in the previsit tool Strokehälsa (English version; reproduced from Kjörk et al [13], which is published under Creative Commons Attribution 4.0 International License [26]).

Strokehealth version 1.0 (Figure 1) began with a brief introductory text. Within this text, patients were encouraged to read additional information on stroke prevention and self-management strategies, with a clickable web link leading to advisory texts. Following the introductory text, patients answered 14 questions related to various health areas. These questions provided three response options, “yes,” “no,” and “choose not to answer,” depending on whether respondents indicated a health problem or not. Finally, at the conclusion of Strokehealth, patients were given the opportunity to provide a free-text response to the question: “Is there anything else you want to add before your visit?”

### Data Collection From the Patient Satisfaction Survey

The survey was constructed in accordance with the technology acceptance model, with a focus on ease of use and perceived usefulness and acceptability [27] regarding Strokehealth. Aspects of ease of use were explored, for example, related to the need for support from others when using Strokehealth, satisfaction with navigating in the tool, and access to the advisory text (web link). Perceived usefulness was explored, for example, related to whether their health problems were captured and their satisfaction with using Strokehealth before a visit. Acceptability was explored, for example, regarding their satisfaction with the layout, answer options, and if they would recommend its use. The usability and technical functionality of the survey were tested in collaboration with a co-designing partner patient before the survey was fielded.

The survey was visible as a 3-page survey containing 15 items with fixed response options and 4 items for free-text answers. The items were divided into different focus areas and each page included 3, 4, and 12 items, respectively, with the ability to scroll before viewing the next page. The items covered demographic data (age, sex, living conditions, education, and source of income), internet use habits, devices used to complete Strokehealth, perceptions about the usability of Strokehealth, and any stroke-related health problem that respondents felt was not addressed. For the last 2 questions, the 5 response options “very satisfied,” “satisfied,” “dissatisfied,” “very dissatisfied,” and “don’t know” were presented with no subsequent adaptive questioning. The 4 items for free-text answers were “Did you miss any health-related problems?”; “Name three advantages of Strokehealth”; “Name three disadvantages of Strokehealth”; and “Do you have any suggestions for improvement?” Before a completed survey was submitted, respondents could view a summary of the responses and change any response if they wished. Once the survey was completed and submitted, respondents could not enter the survey interface again. Patients who filled out Strokehealth but did not complete the subsequent survey could receive a reminder.

### Additional Data Collection

To describe the diagnoses and other characteristics correctly, clinical data were retrieved from the national stroke quality registry Riksstroke. Information included prestroke living conditions, stroke characteristics, and length of hospital stay. If data were missing, complementary information was collected from the medical records (although not all information could be retrieved due to missing data). Furthermore, on a few occasions, it became evident that some respondents did not fully understand one of the questions in the survey. Clarification was then obtained through personal interviews (conducted by EKK).

### Data Analysis

Data were anonymized before all analyses to ensure patient privacy protection. Descriptive statistics were performed using IBM SPSS Statistics (version 24). Categorical values are presented by frequencies and proportion and quantitative variables as medians with ranges or IQRs. With respect to Strokehealth, the time interval from patient notification to registration of a response, as well as the responses themselves, were compiled based on the 3 units. We also conducted an analysis comparing data for those who did and did not respond to Strokehealth. For the nonresponse analysis, we used 2 statistical tests, which were the chi-square test of independence for comparison of 2 categorical variables and the Mann-Whitney *U* test for comparing a categorical with a continuous variable.

Free-text answers were analyzed using a qualitative thematic analysis as outlined by Braun and Clarke [28]. First, we (EKK and PP) analyzed the free-text answers from Strokehealth in an inductive manifest manner with a realist approach to create descriptive themes. In addition, we (KK and EKK) analyzed the free-text answers from the survey using an inductive and latent approach, and the underpinning philosophy was based on a constructionistic approach. The analysis started with the authors reading through the responses several times to become familiar with the data and then discussing underlying meanings and patterns, followed by manual coding. The data were then grouped based on potential themes. The authors reviewed themes several times after discussing them.

## Results

### Participant Characteristics and Responding Approaches

**Figure 2:**
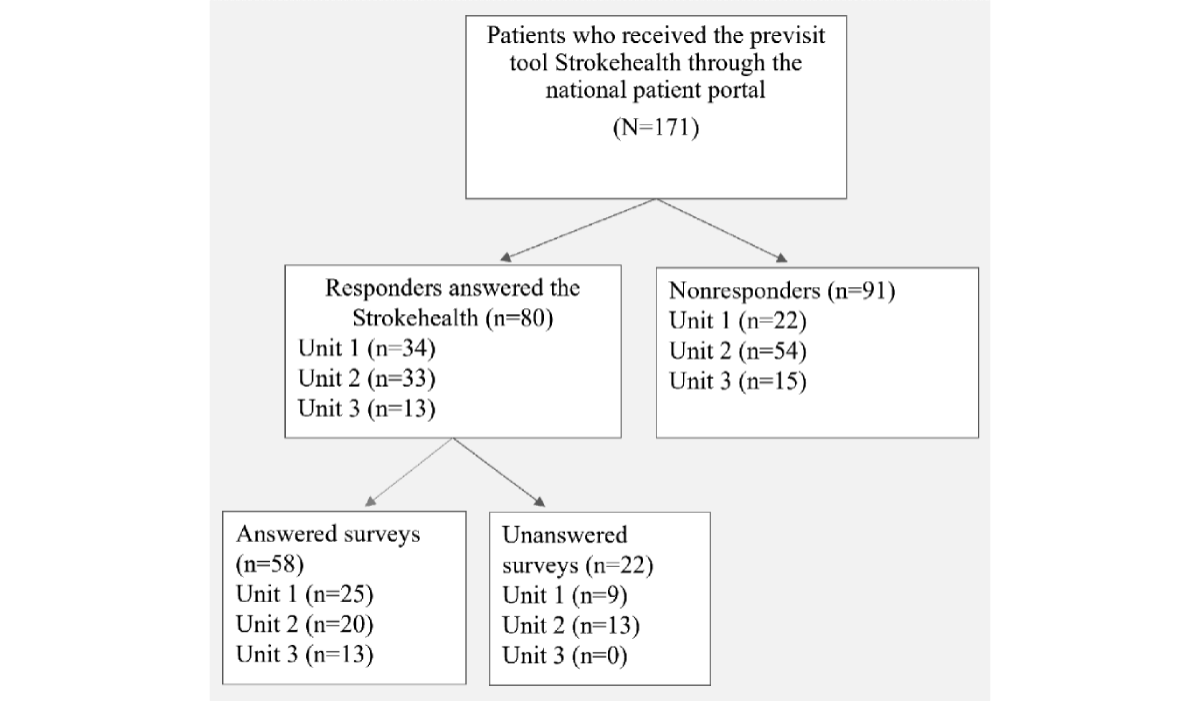
Flowchart of participants and dropouts divided based on the unit where they had their follow-up visit.

In total, 171 people with stroke were consecutively recruited and received the digital previsit tool, Strokehealth. Of these, 40% (68/171) were women, and 60% (103/171) were men. Finally, 47% (80/171) completed Strokehealth, with the largest proportions at unit 1 (34/80, 42%) and unit 2 (33/80, 41%). Patients from unit 1 were younger compared with the other 2 units (Figure 2 and Table 1). The subsequent survey was completed by 73% (58/80; Figure 2). One person started but did not complete the survey, and their responses were not included in the analyses. Most respondents to both Strokehealth (45/80, 56%) and the survey (39/80, 67%) were men. All completed the multiple-choice responses, but only about half responded to one or more of the free-text questions. The most common level of education among respondents was a university degree, and pension compensation was the most prevalent source of income (Table 1).

**Table 1:** Participant characteristics.

Characteristics		Patients answering the previsit tool (n=80)	Respondents of the subsequent survey (n=58)
**Age in years, median (range^a^)**			
	All patients	67 (32-91)	67 (43-91)
	Unit 1	58 (32-85)	57 (43-85)
	Unit 2	71 (44-91)	70 (53-91)
	Unit 3	73 (51-87)	73 (51-87)
**Sex, n (%)**			
	Male	45 (56)	39 (67)
**Education (highest level), n (%)**			
	Mandatory	—^b^	13 (22)
	High School	—	17 (29)
	University	—	25 (43)
	Other	—	3 (5)
**Source of income at inclusion, n (%)**			
	Work	—	24 (41)
	Sick leave	—	3 (5)
	Retirement	—	29 (50)
	Studies	—	1 (2)
	Other	—	1 (2)
**Prestroke living conditions^c^, n (%)**			
	Without assisted care in own home	80 (100)	58 (100)
	Living alone	22 (28)	13 (24)
	Independent	71 (89)	51 (96)
**Stroke characteristics (onset), n (%)**			
	Cerebral infarct	65 (81)	51(87)
	Intracerebral hemorrhage	4 (5)	4 (7)
	Other cerebrovascular events^d^	5 (6)	3 (5)
	Previous stroke	10 (12)	6 (10)
Stroke severity^c^, NIHSS^e^, median (range^a^)		1 (0-13)	1 (0-13)
**Stroke-related outcomes**			
	Length of hospital stay in days, median (range^a^)	5 (1-35)	5 (1-35)
	Discharged to own home, n (%)	80 (100)	58 (100)

^a^Range: minimum-maximum.

^b^Not applicable.

^c^Missing data: Prestroke living conditions (n=5), stroke severity (n=7).

^d^Other cerebrovascular events included transient ischemic attack (n= 3), subarachnoid bleeding (n=1), and sinus thrombosis (n=1).

^e^NIHSS: National Institutes of Health Stroke Scale; measured ≤24 hours of admission, normal values 0-42. Values are presented as numbers and valid percentages unless stated otherwise.

The time interval from patient notification of Strokehealth to registration of a patient response varied greatly, with a median response time of 13 hours (range 9 minutes to 14 days). Internet use was high, with most respondents (48/58, 82%) reporting using the internet several times a day, and others (6/58, 10%) using it a few times a week, a few (2/58, 3%) times each month, or a few times each year (2/58, 3%). The most commonly used device was a smartphone (29/58, 50%), followed by a computer (24/58, 41%) and a tablet such as an iPad (Apple Inc; 5/58, 9%).

### Comparing Patients Responding to the Previsit Tool Strokehealth Versus Nonrespondents

Respondents and nonrespondents differed significantly in age (*P*<.001), with a median age of 67 (IQR 56-75) years among respondents compared with 77 (IQR 69-83) years among nonrespondents. Gender proportions did not differ between the 2 groups (*P*=.32). In comparison with the included patients whose data are given in Table 1, nonrespondents (91/171, 53%) had a higher National Institutes of Health Stroke Scale score (stroke severity), with a median of 2 (IQR 1-6) versus 1 (IQR 0-3), and more often had a history of a previous stroke (17/87, 20%, vs 10/80, 12% for respondents). The proportion with cerebral infarct was 91% (82/90), compared with 81% (65/80) among respondents. Furthermore, 97% (84/87) were discharged to their own home, whereas 100% (80/80) of respondents were discharged home.

### Stroke-Related Health Problems Identified Within the Previsit Tool Strokehealth

**Figure 3:**
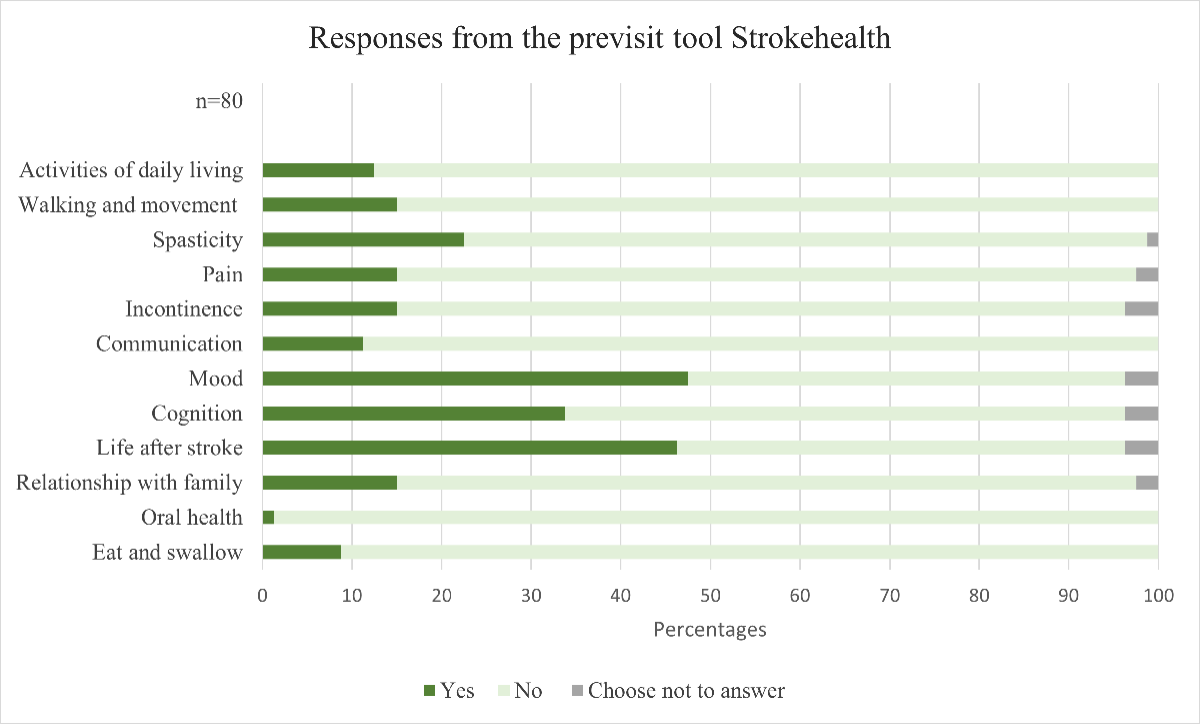
Response displayed by percentage of respondents to the different health areas brought up in the previsit tool Strokehealth.

Among those completing Strokehealth (80/171, the most reported health problems were as follows: mood, with 48% (38/80) experiencing feelings of anxiety or depression after their stroke; life after stroke, with 46% (37/80) noticing difficulties in carrying out tasks they deemed important; and cognition, with 34% (27/80) facing challenges in thinking, concentrating, and remembering. Conversely, oral health was the least frequently reported area, with only 1 respondent indicating a related issue (Figure 3).

The initial question on Strokehealth, about the patient’s interest in receiving information on stroke prevention, received the highest proportion of affirmative responses (70/80, 88%). Patients reported an average of 2 health problems per person (median 2, IQR 0-3.8). When considering different care units, patients at unit 1 had a lower average number of problems (median 1.5, IQR 0-3) compared with patients at unit 2 (median 2, IQR 1-5) and unit 3 (median 2, IQR 0-5.5).

The free-text option in the previsit tool (“Is there anything else you want to add before your visit?”) was used by 45% (36/80) of patients. The thematic qualitative analysis generated 3 categories (Textbox 1).

**Categories created by thematic qualitative analysis based on answers in free-text in the previsit tool Strokehealth.**

**Health- and risk-related worries:**

- questions about medication side effects;
- medical records and requests for advice or practical support;
- concerns about health, including surgery risks; and
- specific questions centered on recommendations for returning to work, medical certificates, and use of a ladder.

**Health problems and their daily impact:**

- descriptions of health issues and perceived impact on daily life activities;
- health problems noted in relation to language, vision, taste, weakness, mobility, sensory perceptions, dizziness, balance, headaches, irritability, and sensitivity to sound and light; and
- impact on daily life related to isolation because of driving limitations and difficulties with house cleaning, baking, taking walks, shopping, and an inability to last the entire day.

**Explanation of personal circumstances:**

- factors that may have influenced their responses or general comments,
- additional diagnoses mentioned such as multiple sclerosis, and
- expressed a desire to provide more nuanced responses to certain questions during the visit.

### Satisfaction With the Previsit Tool

The majority of respondents (56/58, 97%) expressed satisfaction or high satisfaction with how well the previsit tool captured their health problems after stroke. In the free-text option, a few respondents identified some missing health areas in the previsit tool. These included vision (1/58), fatigue (2/58), mental health (1/58), and the location of pain, if present (1/58). The remaining responses to this question (6/58) pertained to the design of the previsit tool rather than to a missing health area.

**Figure 4:**
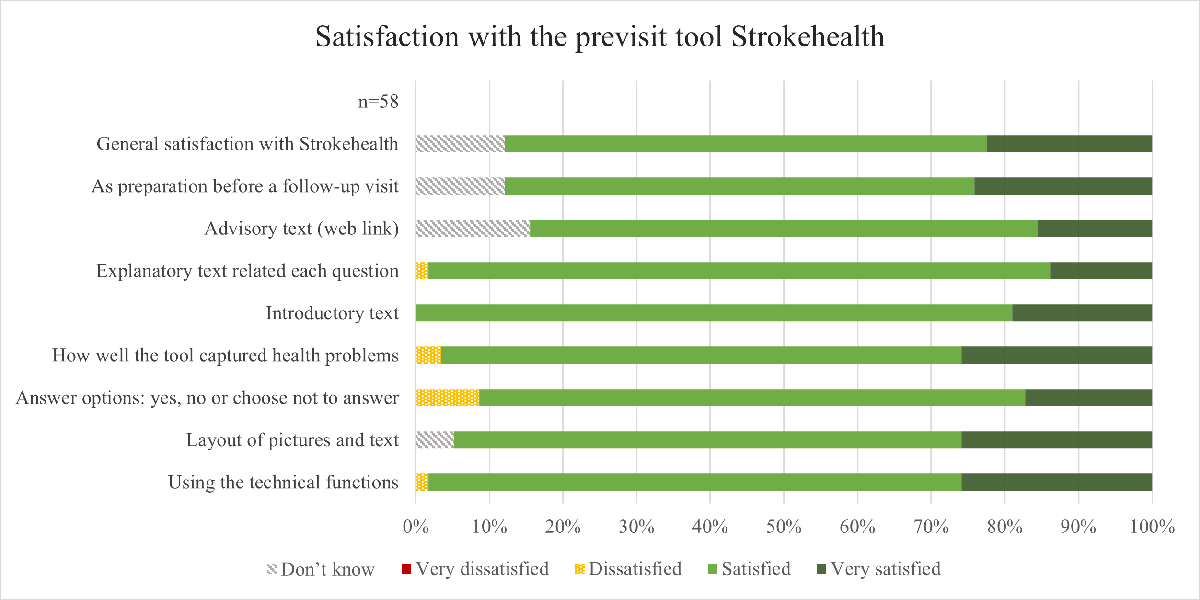
Satisfaction among respondents to the survey regarding different aspects of the previsit tool Strokehealth. Answers are displayed by percentage (n=58). Full formulations of the survey questions can be found in Multimedia Appendix 2.

Overall, the feedback and satisfaction with Strokehealth were positive (Figure 4). None of the 58 individuals reported being very dissatisfied. Similarly, the number of respondents expressing dissatisfaction with any aspect of Strokehealth was minimal. The majority of respondents expressed either satisfaction or high satisfaction on the items addressing their experience with Strokehealth (Figure 4).

A question that received the highest number of “Don’t know” responses (9/58) pertained to satisfaction with the advisory text about self-care and available support resources. This information was provided through a separate link, which some respondents did not see (10/58). The highest level of dissatisfaction (5/58) related to the layout of the response options in Strokehealth (“Yes,” “No,” or “Choose not to answer”).

Most respondents (43/58, 74%) expressed an intention to recommend Strokehealth to others who had experienced a stroke, but 26% (15/58) responded with “Don’t know.” The qualitative interviews (conducted by EKK) clarified that some respondents did not fully understand the question or found it challenging to respond on behalf of someone else. Of note, none of the 58 survey respondents stated that they would not recommend Strokehealth to others who had experienced a stroke.

### Survey: Qualitative Text Analysis

In total, there were 20, 19, 17, and 10 written responses, respectively, to the 4 free-text questions on the survey. After analyzing these answers, the authors developed 4 themes that are described further with illustrative quotations.

#### Structure That Ensures That Issues Will Be Emphasized

Strokehealth was perceived as a valuable tool for structured follow-up care. Respondents expressed that it served as a supportive mechanism, capturing important aspects that might otherwise have gone unnoticed. In addition, respondents believed that Strokehealth facilitated increased involvement in their own care. The tool was reported to provide comprehensive and informative content, offering enhanced insights into their condition and available support. This sentiment is echoed in the following quotes: “Some things that had not been mentioned previously by health professionals were emphasized” and “good and clear debrief.” Furthermore, responses such as “good to prepare oneself in peace and quiet before the conversation” or “It [Strokehealth] helps me to reflect on my situation” indicated that Strokehealth was perceived as a structured tool for reflection and preparation.

#### Importance of User-Friendliness and Accessibility

Statements such as “Good that it is available on the web and easy to access the national patient portal” or “Easy to fill out” indicated that the previsit tool was perceived as user-friendly and readily accessible. However, 1 participant expressed the desire for “Better information than an email that it [Strokehealth] was available to fill out,” suggesting that some individuals may have been unaware of Strokehealth. To enhance usability, 1 participant suggested the ability to reopen and make alterations after completing the tool. In terms of usefulness, patient respondents thought that Strokehealth was a time-saving resource for health professionals. Nevertheless, suggestions for improvement included the desire for prompt feedback on completed responses. Furthermore, one participant’s comment, “Define more what ‘strokehälsa’ is,” may reflect either a lack of sufficient background information about Strokehealth or a misunderstanding of its identity as a digital tool.

#### A Digital Approach as a Means of Communication

Using a digital tool such as Strokehealth can contribute to patients’ feeling more acknowledged and heard. One of the patients stated that 1 advantage of Strokehealth was that “the questions are asked at all and [I] am given the opportunity to be answered.” Another expressed “It feels good to get to answer questions about the stroke.” One of the respondents remarked, “This is the first contact I’ve had with medical care since I was discharged from the stroke unit. I wonder if this is the best contact?” Similarly, another respondent stated, “… Forms, either on paper or digital, create an impersonal impression in an often painful situation. I suppose the spectrum of how this is received by the patients is wide, depending on the consequences of the stroke.” Thus, attitudes toward the use of digital tools and the sense of inclusion or exclusion within the digital context played a significant role in shaping the perception of Strokehealth.

#### Experience of Answering Influenced by Response Options

Respondents frequently mentioned the answer options and a need to explain further and elaborate on their answers. They expressed dissatisfaction with the limited response options of “Yes,” “No,” and “Choose not to answer” for problem areas, as they felt that these options did not adequately capture the complexity of their individual situations. As 1 respondent noted, “The answers to the questions were a bit too much ‘all or nothing’, there weren’t enough alternatives in between.” Participants also highlighted the absence of opportunities to provide explanations or elaborate on their answers, stating that the single free-text question at the end of the tool was insufficient. One participant commented, “If the response was ‘No’, one wanted to be able to explain it in direct connection to that question.” Furthermore, patients complained about the limited word count allowed for the sole free-text response. This limitation also was visible in practice, in that several free-text replies ended abruptly in the middle of a sentence. In addition, respondents found some of the explanatory texts unclear or inconsistent with the questions, leading to confusion and the need to make assumptions about their intended meaning. Furthermore, respondents identified specific missing questions in the tool, such as inquiries about pain localization, while acknowledging the challenge of encompassing every individual’s unique problem within a standardized format.

## Discussion

### Principal Findings and Comparison With Previous Work

The digital previsit tool Strokehealth was designed for patients with stroke to complete before a follow-up visit to support a focused discussion with the stroke team member during the visit. The current findings from this real-world feasibility testing indicate that Strokehealth is a user-friendly and useful tool, essentially confirming previous findings [13]. Furthermore, Strokehealth effectively captured stroke-related health problems and prepared patients satisfactorily for the visit with the health care professional. However, data collected from Strokehealth and the subsequent survey also highlight important aspects to consider in the continuing co-design process to ensure that the tool meets patient needs.

Respondents considered that Strokehealth satisfactorily facilitated the process of identifying stroke-related health problems, even though the vast majority had experienced what was assessed to be a mild stroke. In addition, these findings support that subtle symptoms such as cognitive impairments are common even after clinical recovery from stroke [29]. This study thus confirms the previously recognized potential benefits of a tool like Strokehealth [13]. All the health problems noted in Strokehealth had at least 1 response, demonstrating the relevance of the original Post-Stroke Checklist [9], as well as the 3 additional questions. However, patients also mentioned other health issues in Strokehealth and the survey. These issues are not specified as questions in Strokehealth but can be regarded as indirectly assessed (eg, fatigue, vision, or headache) in a manner analogous to the checklist [10,11]. Furthermore, the high proportion (36/80, 45%) using the free-text option at the end of Strokehealth illustrates that any perceived health issues can be identified in some way with this tool. The free-text option enables each patient’s unique needs to be captured, in keeping with person-centered care [7,8]. Accordingly, the current findings indicate that the number of questions and response options in Strokehealth work satisfactorily in combination with free-text options to identify a patient’s health problems. However, our qualitative findings indicate that the level of answer options, information, and free text needs to be considered (unpublished data) together with the results presented here.

Of note, digital tools such as Strokehealth should be considered an integrated part of the health service [16,21,30]. Our results indicate that Strokehealth has the potential to empower patients and increase their engagement in the follow-up process. In contrast to conventional health care practices in which patients are summoned for prescheduled visits, Strokehealth gives patients the chance to be informed about common consequences after stroke and to reflect on them in advance. The invitation to complete Strokehealth thus can be seen as a starting point for a shared decision-making process, enhancing patient-provider communication during the visit [30] by motivating patients to think about their life after stroke. However, the theme “digital approach as means of communication” raises awareness about patient expectations. Although Strokehealth provided additional support compared with traditional care, some people expected more from their first contact with health care. The principle of “digital first” has become an increasingly common strategy to facilitate proactive support or triage before a physical in-person visit [31], but it may not be consonant with current patient expectations. A patient’s experience with health care relies to a large extent on a health care system’s ability to meet patient expectations [32]. This reliance underscores that the development and use of Strokehealth need to be handled as an integrated part of overall follow-up [21,30] and adapted for the local context [22] (eg, provide information regarding follow-up routines before discharge) to better meet patient expectations.

The fact that 47% (80/171) of the patients filled in Strokehealth in advance is encouraging and shows that they perceive Strokehealth as acceptable. Also encouraging is that most respondents used a smartphone when filling in the form, which suggests that Strokehealth contributes to more accessible health care. The patient is no longer restricted to health care facilities or their own home to take part in health services; instead, they can prepare for a visit at the time and place of their choosing. However, the impact of contextual factors on effectiveness, acceptability [22], and eHealth literacy [19,33] is important to consider. A patient’s ability to engage with digital health services is influenced by how well the system meets patient needs and not only a patient’s ability to understand and use health information [16,19]. Easy access prompts a design in which the patient is asked to fill in Strokehealth in a time and place that supports reflection. Since a few patients submitted Strokehealth very quickly (within 9 minutes), there may be a need to add clarification in the introductory information, for example, a sentence encouraging patients to choose an appropriate time to answer. Understanding the contextual factors influencing these response experiences can provide valuable insights into user’s behavior and preferences, which in turn can inform continuous design and increase accessibility to health care services [21,22]. The initial attempt with Strokehealth [13] to maintain an easy-to-use tool guided by theoretical frameworks (eg, technology acceptance model) [27] with a person-centered focus [7] will also guide the ongoing co-design process.

Strokehealth was perceived to be feasible in a group of people who were almost all using the internet daily. In Sweden, 7 out of 10 people who were older than 75 years use the internet, and many retired people are as accustomed as younger people to doing so [34]. Although patients who completed Strokehealth were statistically significantly younger than those who did not, the number of elderly people using digital services is increasing [34,35]. With this in mind, Strokehealth was co-designed with stakeholders to meet future demands. Others have emphasized the importance of close collaboration with all stakeholders when developing effective digital health services [21]. However, several aspects still must be considered in enhancing user friendliness for the broad range of people with stroke. Among factors to consider in the design process are the ability to process information, the need to feel secure, and patients’ motivations to be engaged in their own care [19]. Although next-of-kin were involved as support in some cases in this study, this involvement might not have been necessary if potential barriers were better addressed. Not all people are motivated or able to use digital health services, and for this reason, Strokehealth is available in multiple languages, in a picture-supported version, and in paper format [36]. Altogether, offering Strokehealth in different modalities is aimed at overcoming certain accessibility challenges to better meet the needs of the broad range of people with stroke.

Overall, this study contributes to the growing body of evidence supporting the effectiveness and implementation of digital tools in health care [21,37,38]. It is now recommended within the Swedish health care system that Strokehealth is administered before a planned follow-up visit and is thus expected to aid health care teams in adopting a previsit planning approach. Consequently, the preparatory opportunities that Strokehealth provides and the time during the actual visit can be used more effectively. Moreover, with increased knowledge, the hope is that patients can reduce their reliance on health care support and rely more on self-management strategies. User-friendly tools indeed have been shown to increase engagement and adherence to self-management strategies [17,39], which is also one of the main purposes of Strokehealth. By building on existing research, future studies can further explore and refine the role of digital tools, such as Strokehealth, in improving the quality of stroke care. More in-depth qualitative investigation with patients as well as with health care professionals is needed to explore the experiences of using Strokehealth, including among diverse social groups and people with communication difficulties or complex needs.

### Strengths and Limitations

A major strength of this feasibility testing is the evaluation of Strokehealth in a clinical context with real world patients before a scheduled follow-up visit. Furthermore, respondents represented people across a range of ages, sexes, education levels, and different health care settings in urban and rural areas. Consequently, the generalizability of the tool’s usability is enhanced. However, there are some limitations. First, despite consecutive sampling, the follow-up routines at each site influenced the sample, leading to a lack of patients with more severe stroke or a higher level of dependency in daily life, and to a range of time intervals since the stroke. Nevertheless, this representation is in line with the stroke population in Sweden, where most patients are classified as having had a mild stroke [40]. Second, during inclusion, researchers did not verify the diagnosis, and some people invited to follow-up had a potential transient ischemic attack that was only recognized later through checks of the health registry or charts. In balance against this limitation, another strength of the study is the comprehensive description of clinical and demographic data, which in turn provides knowledge about the potential target group for Strokehealth. Third, the influence of recall bias cannot be ruled out for the responses to Strokehealth or the survey. Given the number of free-text answers, though, it seems that most respondents had a clear opinion. A final strength is the high response rate of the survey. Since most strokes are mild in nature, as confirmed in our study, the current version of Strokehealth would be considered a user-friendly tool for most people with stroke.

### Conclusions

This real-world feasibility study shows that the previsit tool, Strokehealth, is feasible when used before a follow-up visit after a stroke. Satisfaction with the tool’s ability to capture health problems was high among patients, with a majority having experienced a mild stroke and being regular users of the internet. However, during subsequent co-design processes, features such as free-text options and information need to be considered in parallel with qualitative data. Further research is needed to explore the use and benefits for a broader range of users, including people with communication difficulties, and to gain health professionals’ perspectives.

## Appendix

Multimedia Appendix 1 CHERRIES (Checklist for Reporting Results of Internet E-Surveys) checklist.

Multimedia Appendix 2 Patient satisfaction survey translated into English.

## Abbreviations

CHERRIES: Checklist for Reporting Results of Internet E-Surveys
