# Supplementary material for: Evaluation of a Digital Previsit Tool for Identifying Stroke-Related Health Problems Before a Follow-Up Visit (Part 1): Survey Study
Source: JMIR Hum Factors. 2024 Sep 3;11:e55852. doi: 10.2196/55852 (PMC11408892; doi:10.2196/55852)
Supplement: Multimedia Appendix 2 [file humanfactors_v11i1e55852_app2.docx]

**Structure for the Web Survey About the Previsit Tool Strokehälsa**

**Background questions**

We asked the following questions, answer options are listed in brackets (most were mandatory):

- How often do you use the internet via computer/mobile phone or tablet?
  (Never; Sometime a year; Sometime a month; Sometime a week; or Several times a day)
- How did you answer the questions in Strokehälsa?
  (Computer; Mobile phone; Tablet (such as iPad); or Other unit)
- When you filled in Strokehälsa, did someone assist you? (Yes; or No)
- What is your highest completed level of education?
  (Mandatory; High school; University; or Other)
- What is your main source of income?
  (Work; Sick leave; Retirement; Unemployment compensation; Studies; or Other)

**When you think back to what it was like to complete Strokehälsa:**

- How satisfied are you with marking your answers and progressing in Strokehälsa?
  (Very satisfied; Satisfied; Dissatisfied; Very dissatisfied; or Don’t know)
- How satisfied are you with the layout (appearance of image and text)?
  (Very satisfied; Satisfied; Dissatisfied; Very dissatisfied; or Don’t know)
- How satisfied are you with the response options: Yes / No / Choose not to answer?
  (Very satisfied; Satisfied; Dissatisfied; Very dissatisfied; or Don’t know)

**When you think back on your experience using Strokehälsa:**

- How satisfied are you with how well the questions captured your health problems after your stroke? (Very satisfied; Satisfied; Dissatisfied; Very dissatisfied; or Don’t know)
- Was there a health problem you missed? (Free-text space)
- How satisfied were you with the initial information* at the beginning of Strokehälsa?
  (Very satisfied; Satisfied; Dissatisfied; Very dissatisfied; or Don’t know)
  * *referred to as ‘introductory text’ in the paper*
- How satisfied were you with the information in connection with the questions**?
  (Very satisfied; Satisfied; Dissatisfied; Very dissatisfied; or Don’t know)
  ** *referred to as ‘explanatory text’ in the paper*
- Have you read the information provided about self-care and support***, accessible via the link at the end of the form? (Yes; or No)
  *** *referred to as ‘advisory text’ in the paper*
- How satisfied are you with the information about what you can do yourself and how you can get support? (Very satisfied; Satisfied; Dissatisfied; Very dissatisfied; or Don’t know)
- How satisfied are you with using Strokehälsa as a preparation for your healthcare visit?
  (Very satisfied; Satisfied; Dissatisfied; Very dissatisfied; or Don’t know)
- In general, how satisfied are you with Strokehälsa?
  (Very satisfied; Satisfied; Dissatisfied; Very dissatisfied; or Don’t know)
- Would you recommend Strokehälsa to someone who has had a stroke?
  (Yes; No; I doubt it; or Don’t know)
- Name three benefits of Strokehälsa. (Free-text space)
- Name three disadvantages of Strokehälsa (Free-text space)
- Do you have suggestions for improvements? (Free-text space)

**Summary**

Read through your answers and confirm by clicking the “Confirm your answers” button at the bottom of this page.

[A table appears with a summary of the questions and the chosen options. A clickable pen is visible at each question if a patient wishes to change the answer before submitting the survey]
